# Supplementary material for: miR-27b attenuates apoptosis induced by transmissible gastroenteritis virus (TGEV) infection via targeting runt-related transcription factor 1 (RUNX1)
Source: PeerJ. 2016 Feb 4;4:e1635. doi: 10.7717/peerj.1635 (PMC4748701; doi:10.7717/peerj.1635)
Supplement: Table S1 [file peerj-04-1635-s001.doc]

**Supplementary Table S1**

**Sequences of primer pairs used for gene of RUNX1.**

| Plasmid | Forward primer (5'-3') | Reverse primer (5'- 3') |
| --- | --- | --- |
| psiCHECH2-RUNX1-WT | CCGCTCGAGAGATTTTTCAAACCTGACGCA | ATTTGCGGCCGCAAAATAAAAC CACCCCAAATG |
| psiCHECH2-RUNX1- mut | TCCCAGAGGGAAAATCTCAATGCTTCTGATTTAGCAATGATCTCAATAAAAGAAAGATT | AATCTTTCTTTTATTGAGATCATTGCTAAATCAGAAGCATTGAGATTTTCCCTCTGGGA |
| pCI-neo-RUNX1 | CCGGAATTCATGGCTTCAGACAGCATATTTGAGT | ATTTGCGGCCGCTCAGTAGGGCCGCCATACG |
