# Supplementary material for: miR-27b attenuates apoptosis induced by transmissible gastroenteritis virus (TGEV) infection via targeting runt-related transcription factor 1 (RUNX1)
Source: PeerJ. 2016 Feb 4;4:e1635. doi: 10.7717/peerj.1635 (PMC4748701; doi:10.7717/peerj.1635)
Supplement: Table S2 [file peerj-04-1635-s002.doc]

**Supplementary Table S2**

**Sequences of** miRNA and siRNAs

| Small RNA | Sense strand (5'-3') | antisense strand (5'- 3') |
| --- | --- | --- |
| miR-27b mimics | UUCACAGUGGCUAAGUUCUGC | GCAGAACUUAGCCACUGUGAA |
| miRNA mimics control | UCACAACCUCCUAGAAAGAGUAGA | UCUACUCUUUCUAGGAGGU UGUGA |
| miR-27b inhibitors | GCAGAACUUAGCCACUGUGAA |  |
| miRNA inhibitors control | UCUACUCUUUCUAGGAGGUUGUGA |  |
| si-RUNX1-2 | GAACCACUCCACUGCCUUUdTdT | AAAGGCAGUGGAGUGGUUCdTdT |
