# Supplementary material for: miR-27b attenuates apoptosis induced by transmissible gastroenteritis virus (TGEV) infection via targeting runt-related transcription factor 1 (RUNX1)
Source: PeerJ. 2016 Feb 4;4:e1635. doi: 10.7717/peerj.1635 (PMC4748701; doi:10.7717/peerj.1635)
Supplement: Table S3 [file peerj-04-1635-s003.doc]

**Supplementary Table S3**

**Sequences of primer pairs used for Real-time PCR.**

| Gene | Forward primer (5'-3') | Reverse primer (5'- 3') |
| --- | --- | --- |
| RUNX1 | AACCCTCAGCCTCAGAGTCA | GCAATGGATCCCAGGTATT |
| Bax | ATGATCGCAGCCGTGGA | GGGCCTTGAGCACCAGTTT |
| β-actin | GGACTTCGAGCAGGAGATGG | AGGAAGGAGGGCTGGAAGAG |
