# Supplementary figures and images for: miR-27b attenuates apoptosis induced by transmissible gastroenteritis virus (TGEV) infection via targeting runt-related transcription factor 1 (RUNX1)
Source: PeerJ. 2016 Feb 4;4:e1635. doi: 10.7717/peerj.1635 (PMC4748701; doi:10.7717/peerj.1635)

24 hpi

48 hpi

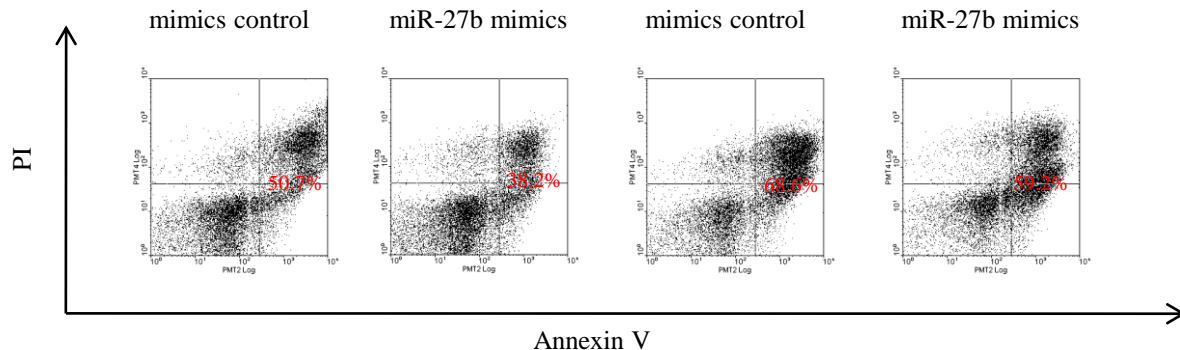

24 hpi

48 hpi

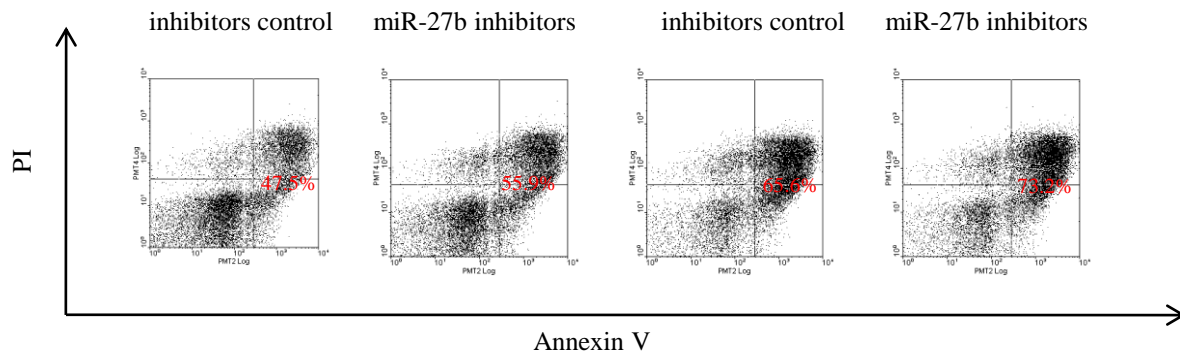

Supplement: Supplemental Information 1 — Figure 1A effect of the miR-27b on apoptosis via flow cytometry. [file peerj-04-1635-s004.pdf]

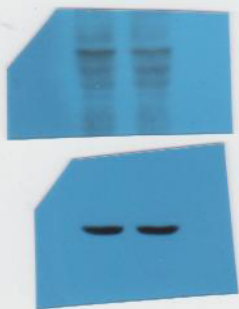

Supplement: Supplemental Information 4 — Fig. 2D Western blot analysis of RUNX1 in cells transfected with miR-27b mimics or mimics control. [file peerj-04-1635-s007.pdf]

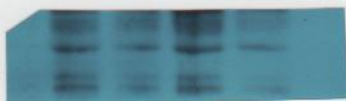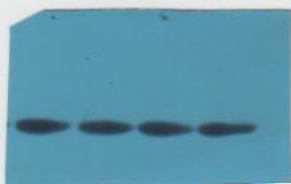

Supplement: Supplemental Information 5 — Fig. 3A1 western blot analysis of Bax in cells transfected with miR-27b mimics. [file peerj-04-1635-s008.pdf]

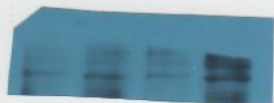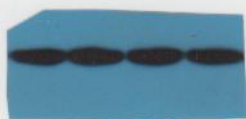

Supplement: Supplemental Information 6 — Fig. 3A2 western blot analysis of Bax in cells transfected with miR-27b inhibitors. [file peerj-04-1635-s009.pdf]

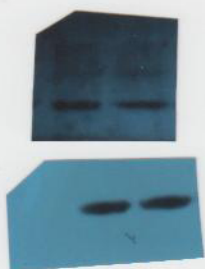

Supplement: Supplemental Information 9 — Figure 4B the silencing effect of siRUNX1-2 on RUNX1 expression. [file peerj-04-1635-s012.pdf]

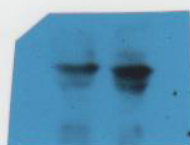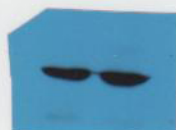

Supplement: Supplemental Information 11 — Figure 4D the over-expression of RUNX1 using pCI-neo-RUNX1. [file peerj-04-1635-s014.pdf]

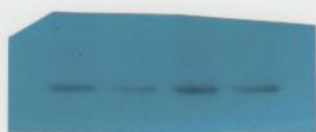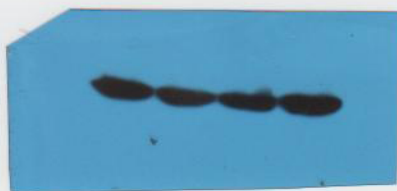

Supplement: Supplemental Information 12 — Figure 4E1 the effect of RUNX1 on the expression of Bax using siRNA. [file peerj-04-1635-s015.pdf]

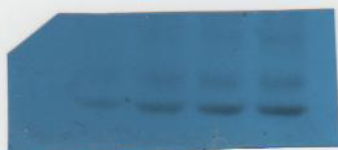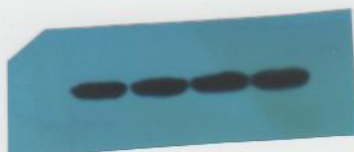

Supplement: Supplemental Information 13 — Figure 4E2 the effect of RUNX1 on the expression of Bax using pCI-neo-RUNX1. [file peerj-04-1635-s016.pdf]
