# Supplementary material for: miR-27b attenuates apoptosis induced by transmissible gastroenteritis virus (TGEV) infection via targeting runt-related transcription factor 1 (RUNX1)
Source: PeerJ. 2016 Feb 4;4:e1635. doi: 10.7717/peerj.1635 (PMC4748701; doi:10.7717/peerj.1635)
Supplement: Supplemental Information 15 [file peerj-04-1635-s018.pdf]

Data not shown

A

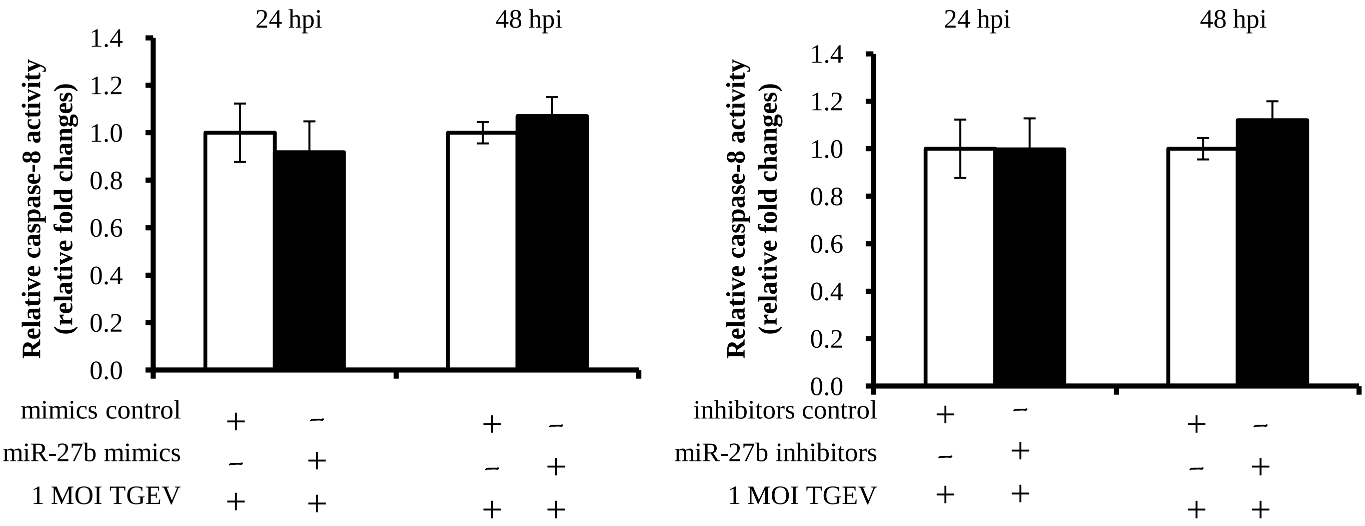

miR-27b did not affect the caspase-8 activity during TGEV infection

B

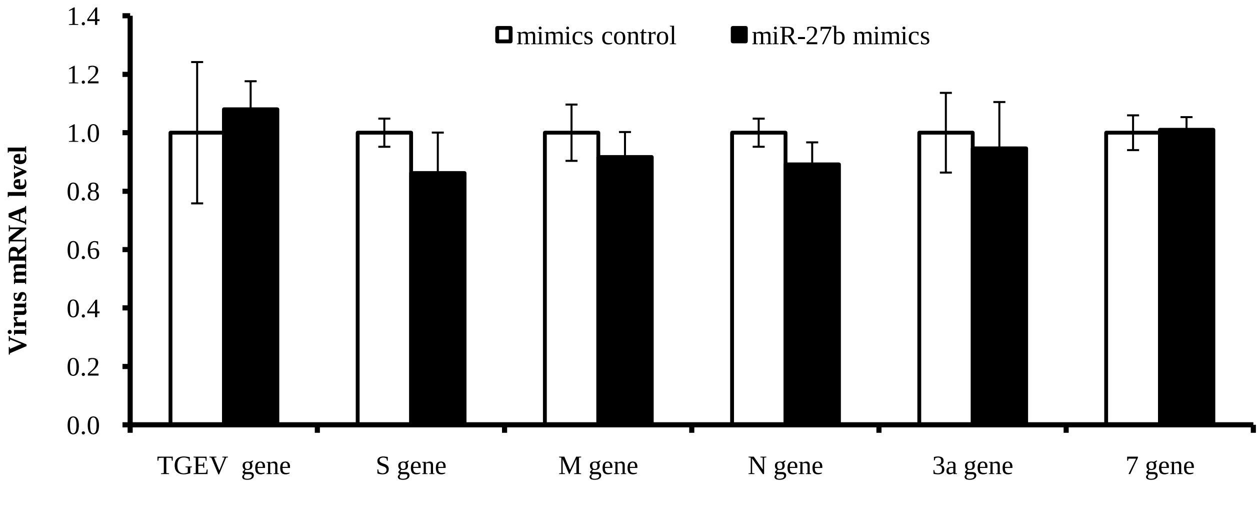

The effects of miR-27b mimics on the replication and transcription of TGEV, the replication and transcription of TGEV structural and non-structural genes were not affected by miR-27b.
